# Recombinant Canine Coronaviruses in Dogs, Europe

**DOI:** 10.3201/eid1601.090726

**Published:** 2010-01

**Authors:** Nicola Decaro, Viviana Mari, Gabriella Elia, Diane D. Addie, Michele Camero, Maria Stella Lucente, Vito Martella, Canio Buonavoglia

**Affiliations:** Faculty of Veterinary Medicine of Bari, Valenzano, Italy (N. Decaro, V. Mari, G. Elia, M. Camero, M.S. Lucente, V. Martella, C. Buonavoglia); Feline Institute Pyrenees, Etchebar, France (D.D. Addie)

**Keywords:** Canine coronavirus, recombinant strains, Europe, genetic analysis, research

## Abstract

Coronaviruses of potential recombinant origin with porcine transmissible gastroenteritis virus (TGEV), referred to as a new subtype (IIb) of canine coronavirus (CCoV), were recently identified in dogs in Europe. To assess the distribution of the TGEV-like CCoV subtype, during 2001–2008 we tested fecal samples from dogs with gastroenteritis. Of 1,172 samples, 493 (42.06%) were positive for CCoV. CCoV-II was found in 218 samples, and CCoV-I and CCoV-II genotypes were found in 182. Approximately 20% of the samples with CCoV-II had the TGEV-like subtype; detection rates varied according to geographic origin. The highest and lowest rates of prevalence for CCoV-II infection were found in samples from Hungary and Greece (96.87% and 3.45%, respectively). Sequence and phylogenetic analyses showed that the CCoV-IIb strains were related to prototype TGEV-like strains in the 5′ and the 3′ ends of the spike protein gene.

## Abstract 2

Subtype IIb originates from recombination with porcine transmissible gastroenteritis virus.

Coronaviruses (CoVs) (order Nidovirales, family *Coronaviridae*) are exceptionally prone to genetic evolution through accumulation of point mutations in genes encoding for structural and nonstructural proteins and homologous recombination among members of the same antigenic group (*1*). CoVs are organized by antigenic group. The first group is subdivided into subgroups 1a and 1b. Subgroup 1a includes highly related viruses (i.e., porcine transmissible gastroenteritis virus [TGEV] and its derivative porcine respiratory coronavirus [PRCoV], feline coronaviruses [FCoVs], and canine coronaviruses [CCoVs]) (*2*). According to a proposal by the Coronavirus Study Group of the International Committee of Taxonomy of Viruses, and given the virus’ close genetic relatedness (i.e., >96% aa identity in the key replicase 1ab domains), TGEV, FCoV, and CCoV should not be considered separate viruses. Instead, they should be considered host range variants of the same species (*3*).

CCoVs exemplify the genetic evolution and complexity of CoVs. To date, 2 CCoV genotypes are known, CCoV-I and CCoV-II (*4*); they share up to 96% of nucleotide identity in the viral genome (Lorusso et al., unpub. data) but are highly divergent in the spike protein gene (*5*). In addition, CCoV-I displays a novel open reading frame (ORF3) that encodes for a putative glycosylated protein, which is likely secreted from the infected cells (*6*). The 2 CCoV genotypes are commonly detected in the feces of dogs with diarrhea and often simultaneously infect the same dog (*7*). Both CCoV genotypes have been associated with mild clinical signs in pups, although hypervirulent strains have been reported to cause severe, fatal enteritis (*8*–*10*); a pantropic variant was responsible for systemic disease in natural and experimental conditions (*11*–*13*).

It has been postulated that TGEV originated from CCoV-II through cross-species transmission, which is supported by the high genetic relatedness between the 2 viruses and by the presence of ORF3 remnants in CCoV-II and TGEV genomes (*6*). More recently, novel CCoV-II strains, which likely originated from a double recombination event with TGEV, occurring in the 5′ end of the spike protein gene, have been isolated (*14*,*15*). Accordingly, genotype II has been further divided into 2 subtypes, CCoV-IIa and CCoV-IIb, including extant and TGEV-like CCoVs, respectively (*14*).

To assess the distribution of the TGEV-like CCoVs in canine populations of different geographic areas of Europe, we used a subtype IIb-specific reverse transcription–PCR (RT-PCR) assay. In addition, we evaluated the genetic relationship among the identified strains by using sequence and phylogenetic analyses.

## Methods

### Sample Origin

During 2001–2008, a total of 1,172 fecal samples were collected from dogs with acute enteritis in 14 European countries: Italy (n = 760), United Kingdom (n = 199), Greece (n = 81), Hungary (n = 41), Portugal (n = 33), Spain (n = 32), Belgium (n = 7), Romania (n = 6), Bulgaria (n = 3), Germany (n = 3), Sweden (n = 3), Slovakia (n = 2), Poland (n = 1), and Slovenia (n = 1). Some samples were retrieved from previous studies (*7*,*16*–*18*), whereas the remaining samples were sent to our laboratory (from veterinarians, breeders, or other researchers) for routine diagnostic investigations.

### RNA Extraction

Specimens were homogenized (10% wt/vol) in Dulbecco modified Eagle medium and subsequently clarified by centrifuging at 2,500 × *g* for 10 min. For RNA extraction, 140 μL of the supernatants were then used by means of QIAamp Viral RNA Mini Kit (QIAGEN S.p.A., Milan, Italy); according to the manufacturer’s protocol, RNA templates were stored at –70°C until use.

### CCoV RNA Detection, Quantification, and Genotyping

For rapid detection and quantification of CCoV RNA, all RNA extracts were subjected to a previously established TaqMan-based real-time RT-PCR (*16*) with minor modifications. Briefly, a 1-step method was adopted by using the Platinum Quantitative PCR ThermoScript One-Step System (Invitrogen S.R.L., Milan, Italy) and the following 50-µL mixture: 25 µL of master mix, 300 nM of primers CCoV-forward (5′-TTGATCGTTTTTATAACGGTTCTACAA-3′) and CCoV-reverse (5′-AATGGGCCATAATAGCCACATAAT-3′), 200 nM of probe CCoV-Pb (5′-FAM-ACCTCAATTTAGCTGGTTCGTGTATGGCATT-BHQ1-3′), and 10 μL of template RNA. To obtain a standard curve for absolute quantification, we simultaneously analyzed duplicates of log_10_ dilutions of standard RNA (*16*). The thermal profile consisted of reverse transcription at 50°C for 20 min, activation of Platinum Taq DNA polymerase at 95°C for 2 min, 45 cycles of denaturation at 95°C for 15 s, annealing at 48°C for 30 s, and extension at 60°C for 30 s.

The positive samples were characterized by 2 distinct genotype-specific assays (*17*) performed by using the Platinum Quantitative PCR ThermoScript One-Step System (Invitrogen S.R.L.) and the following oligonucleotide sets (final concentrations were 600 and 200 nM for primers and probes, respectively): primer pair CCoVI-F (5′-CGTTAGTGCACTTGGAAGAAGCT-3′)/CCoVI-R (5′-ACCAGCCATTTTAAATCCTTCA-3′) and probe CCoVI-Pb (5′-FAM -CCTCTTGAAGGTACACCAA-TAMRA-3′) for CCoV-I; primer pair CCoVII-F (5′-TAGTGCATTAGGAAGAAGCT-3′)/CCoVII-R (5′-AGCAATTTTGAACCCTTC-3′) and probe CCoVII-Pb (5′-FAM-CCTCTTGAAGGTGTGCC-TAMRA-3′) for CCoV-II. The thermal protocol was as described for CCoV detection except for different annealing temperatures (i.e., 53°C and 48°C for CCoV-I and CCoV-II, respectively).

### Development of RT-PCRs Specific for Classical and TGEV-like CCoVs

Considering the high divergence observed in the 5′ end of the spike gene between classical (subtype IIa) and TGEV-like (subtype IIb) CCoVs, specific CCoV-IIa and CCoV-IIb gel-based RT-PCRs were developed. Primer 20179 (sense, 5′-GGCTCTATCACATAACTCAGTCCTAG-3′) binds a conserved region at the 3′ end of ORF1b and was recruited from a previous study (*13*), whereas antisense primers INS-R-dg (5′-GCTGTAACATAKTCRTCATTCCAC-3′) and 174-268 (5′-CAACATGTAACCTTTGTCTGTGATCTGC-3′) target regions at the 5′ end of the spike protein gene of feline CoV-II (FCoV-II)/classical CCoV-II and TGEV/TGEV-like CCoV, respectively. Separate RT-PCRs with primers 20179/INS-R (CCoV-IIa) or 20179/174-268 (CCoV-IIb) were conducted by using SuperScript One-Step RT-PCR for Long Templates (Invitrogen S.R.L.), according to the manufacturer’s instructions. The following thermal protocol was used: reverse transcription at 50°C for 30 min, inactivation of Superscript II RT at 94°C for 2 min, 40 cycles of 94°C for 30 s, 55°C for 30 s, 68°C for 30 s, and final extension at 68°C for 10 min. The PCR products were detected by using electrophoresis through a 1.5% agarose gel and examination under UV light after ethidium bromide staining.

### RT-PCR for Amplification of the 3′ End of the Spike Protein Gene of CCoV-II

To rule out any potential infection by true TGEV strains and to confirm the recombinant origin of the TGEV-like CCoVs, we submitted 20 samples that were positive for CCoV-IIb and negative for CCoV-I to RT-PCR amplification of the 3′ end of the spike protein gene of CCoV-II (*7*). Primers S5 (5′-TGCATTTGTGTCTCAGACTT-3′) and S6 (5′-CCAAGGCCATTTTACATAAG-3′) were used in the RT-PCR, performed according to the protocol described for CCoV subtyping.

### RT-PCR of the ORF7a/7b Region

To rule out the presence of true TGEV strains in the dog feces that were positive by CCoV-IIb–specific assay, we used an RT-PCR that had been proven to discriminate between TGEV and CCoV on the basis of amplicon size (*19*). In fact, primers N3SN (5′-GTGTTTGATGACACACAGGTTGAG-3′) and R3AS (5′-GCTTACCATTCTGTACAAGAGGTAG-3′) target the 3′ end of the viral genome, where CCoV/FCoV and TGEV display 2 (ORFs 7a and 7b) and 1 (ORF7) accessory genes, respectively. As controls, the following reference group-1a CoVs were used: TGEV-Purdue (kindly provided by P. Cordioli, Istituto Zooprofilattico Sperimentale di Lombardia ed Emilia Romagna, Brescia, Italy), FCoV-I-249/04 (*20*), FCoV-II-29/92 (*21*), CCoV-I-Elmo/02 (*5*), CCoV-IIa-CB/05 (*11*), CCoV-IIb-341/05, CCoV-IIb-174/06, CCoV-IIb-430/07, and CCoV-IIb-119/08 (*14*).

### Sequence and Phylogenic Analyses

The RT-PCR products obtained with primer pairs 20179/174-268 and S5/S6 from 26 samples having positive CCoV-IIb–specific assay results and being representative of the different geographic areas were subjected to direct sequencing at the BaseClear B.V. (Leiden, the Netherlands). The sequences were manually edited and analyzed by using BioEdit software (*22*) and National Center for Biotechnology Information (www.ncbi.nlm.nih.gov) and European Molecular Biology Laboratory (www.ebi.ac.uk) analysis tools. Phylogenetic and molecular evolutionary analyses were conducted by using Mega 4.1, beta (*23*). Phylogenetic trees on the basis of partial 5′ (339-nt) and 3′ (520-nt) ends of the spike protein gene were elaborated by using parsimony and neighbor-joining methods, which supplied statistical support with bootstrapping >1,000 replicates. Group-2 CoV canine respiratory CoV-240/05 (*24*) was used as an outgroup. The nucleotide sequences of the analyzed CCoV-IIb strains were deposited in GenBank under accession nos. GQ130243–GQ130268 and GQ148749–GQ148774 for 5′ and 3′ ends of the spike gene, respectively.

## Results

### CCoV Detection and Quantification

CCoV RNA was detected in 493 (42.06%) of 1,172 fecal samples from dogs with diarrhea. Viral RNA titers ranged from 1.25 × 10^1^ to 7.56 × 10^7^ copies/μL of template. For each geographic region of origin, detection rates of the CCoV real-time RT-PCR were Italy 330/760 (43.42%), United Kingdom 54/199 (27.13%), Greece 45/81 (55.5%), Hungary 32/42 (78.05%), Portugal 12/33 (36.36%), Spain 2/32 (6.25%), Belgium 4/7 (57.14), Romania 4/6 (66.66%), Bulgaria 1/3 (33.33%), Germany 3/3 (100%), Sweden 3/3 (100%), Slovakia 1/2 (50%), Poland 1/1 (100%), and Slovenia 1/1 (100%) (Table 1).

**Table 1:** Distribution of CCoV genotypes and TGEV-like strains (CCoV-IIb) in dogs in European countries, 2001–2008*

Country	No. samples	No. or % positive					
		CCoV, no.	CCoV-I no.†	CCoV-II, no.†	CCoV-I+II, no.‡	CCoV-IIb, no.	CCoV-IIb/II, %
Italy	760	330	54	138	138	34	12.32
United Kingdom	199	54	13	24	17	10	24.39
Greece	81	45	16	15	14	1	3.45
Hungary	41	32	0	30	2	31	96.87
Portugal	33	12	6	3	3	0	0
Spain	32	2	0	1	1	0	0
Belgium	7	4	1	3	0	0	0
Romania	6	4	1	1	2	1	33.33
Bulgaria	3	1	0	1	0	0	0
Germany	3	3	0	0	3	0	0
Sweden	3	3	1	1	1	1	50
Slovakia	2	1	0	0	1	0	0
Poland	1	1	0	1	0	0	0
Slovenia	1	1	1	0	0	0	0
Total	1,172	493	93	218	182	78	19.50

*CCoV, canine coronavirus; TGEV, porcine transmissible gastroenteritis virus. †Infections with a single CCoV genotype. ‡Infections with both CCoV genotypes.

### CCoV Genotype and Subtype Distribution

The geographic distribution of the CCoV types and subtypes is reported in Table 1. Genotype-specific amplification assays showed that 93 (18.86%) of 493 CCoV-positive samples were positive for CCoV-I, and 218 (44.22%) were positive for CCoV-II. In addition, 182 samples (36.92%) were positive for both genotypes. Both genotypes were found to circulate in most European countries that had been sampled, and an overall prevalence of CCoV-II was found in all countries except Greece, where the 2 genotypes were detected approximately to the same extent.

By using the developed TGEV-like RT-PCR, we found that 78 (19.50%) of 400 samples containing CCoV-II strains, alone or in combination with CCoV-I, were positive for CCoV-IIb. The remaining 322 CCoV-II strains were positive for subtype IIa, whereas mixed infections caused by both CCoV-II subtypes were not detected in any samples. Almost all CCoV–IIb-positive samples were from Italy (34/276; 12.32%), the United Kingdom (10/41; 24.39%), and Hungary (31/32; 96.87%). Single CCoV-IIb strains were detected in samples from Greece (1/29; 3.45%), Romania (1/3; 33.33%), and Sweden (1/2; 50%), whereas no samples from the other countries had TGEV-like CCoVs.

### RT-PCR of ORF7a/7b Region

After RT-PCR with primer pair N3SN/R3AS, CCoV and FCoV reference strains yielded an amplicon >1,000 bp, with the exception of TGEV-like CCoV 341/05, which gave a 929-bp product as a consequence of a 154-nt deletion in ORF7b (*14*). In contrast, a 367-bp product was obtained from TGEV-Purdue, as previously described (*19*). This pattern of amplification agreed with the absence of ORF7b in TGEV (*25*). All 20 samples that were positive according to the CCoV-IIb–specific assay and negative according to the CCoV-I TaqMan assay were confirmed to contain true CCoV strains because they yielded RT-PCR products considerably larger than the 367-bp amplicon obtained from TGEV.

### Sequence and Phylogenetic Analyses

We selected the following TGEV-like strains for sequence analysis of the 5′ and 3′ ends of the spike protein gene: 12 strains from Italy, 8 from the United Kingdom, and 5 from Hungary, plus the single strains from Greece, Romania, and Sweden. All RT-PCR products were sequenced except those obtained from the samples from Greece and Sweden, which yielded weak bands despite the considerable viral RNA titers in the original fecal samples (5.02 × 10^4^ and 5.76 × 10^6^ RNA copies/μL of template, respectively).

The obtained sequences were compared with each other (Table 2) and with 3 CCoV-IIa, 4 CCoV-IIb, 2 FCoV-II, 2 CCoV-I, 3 FCoV-I, and 3 TGEV reference sequences (Table 3). Sequence comparison of the TGEV-like strains showed overall nucleotide identity of 83.6%–99.6% and 92.7%–100% in the 5′ and 3′ ends of the spike gene, respectively. By analyzing the TGEV-like strains by country of origin, we found the highest genetic variability among the viruses from Italy (86.8%–99.3% and 94.5%–99.8% of nucleotide identity in the gene 5′ and 3′ ends, respectively), whereas the strains from Hungary showed the highest relatedness (92.7%–99.3% and 98.4%–99.6% of nucleotide identity in the gene 5′ and 3′ ends, respectively) (Table 2). The TGEV-like strains exhibited the best identity to prototype strains (*14*) in both the 5′ (85.9%–99.3%) and the 3′ end (92.7%–99.8%) of the spike gene, whereas a slightly lower identity was found to the old strain UCD1 (*19*) in the 5′ end, which is the only sequence available in the GenBank database. When the 5′ end was analyzed, the identified TGEV-like CCoVs were more related to classical TGEVs (76.6%–84.4%) than to type IIa CCoVs (34.2%–38.3%). In contrast, analysis of the 3′ end of the spike gene showed nucleotide identities of 87.1%–93.1% to TGEV and of 90.8%–99.0% to CCoV-IIa. The best identities among CCoV-IIa isolates were to strain Insavc-1, which has been proposed as intermediate virus between CCoV and TGEV (*26*) and to the more recent pantropic strain CB/05 (*11*) in the 5′ and 3′ ends, respectively.

**Table 2:** Percent nucleotide identities in the 5′ (top right) and 3′ (bottom left) ends of the spike gene of CCoV-IIb (TGEV-like) strains from dogs in Europe, 2001–2008*

Country	Italy	United Kingdom	Hungary	Romania	Overall
Italy	86.8–99.3 94.5–99.8	83.6–92.7	86.5–99.0	89.1–99.3	83.6–99.3
United Kingdom	92.7–97.0	88.5–99.6 97.6–99.8	87.8–97.0	87.8–93.1	83.6–99.6
Hungary	94.7–100	92.9–94.9	92.7–99.3 98.4–99.6	94.7–99.3	86.5–99.3
Romania	95.3–99.6	93.5–94.5	98.6–99.6	ND	87.8–99.3
Overall	92.7–100	92.7–99.8	92.9–100	93.5–99.6	83.6–99.6 92.7–100

*CCoV, canine coronavirus; TGEV, porcine transmissible gastroenteritis virus; ND, not done because only a single strain from Romania was analyzed.

**Table 3:** Percent nucleotide identity of representative TGEV-like CCoVs to group-1a CoV reference strains in the 5′ and 3′ ends of the spike gene from dogs from Europe, 2001–2008*

CoV strain	GenBank accession no.	% Identity	
		5′ end†	3′ end‡
CCoV-IIb-341/05	EU856361	88.1–99.3	94.7–99.4
CCoV-IIb-174/06	EU856362	86.8–99.0	93.3–97.0
CCoV-IIb-430/07	EU924790	85.9–97.3	92.7–97.0
CCoV-IIb-119/08	EU924791	89.1–99.0	93.7–99.8
CCoV-IIb-UCD1	AF116248	76.6–85.7	NA
TGEV-Purdue	NC_002306	76.6–84.4	91.4–93.1
TGEV-TS	DQ201447	76.2–84.4	90.8–92.5
TGEV-96-1933	AF104420	74.0–81.8	87.1–88.8
PRCoV-RM4	Z24675	NA	90.2–91.6
PRCoV-86-137004	X60089	NA	90.2–91.6
CCoV-IIa-Insavc-1	D13096	35.4–38.3	92.1–95.3
CCoV-IIa-CB/05	DQ112226	35.1–37.7	93.7–99.0
CCoV-IIa-BGF10	AY342160	34.2–37.5	90.8–94.3
FCoV-II-79-1146	NC_007025	34.0–36.6	90.2–91.6
FCoV-II-79-1683	X80799	34.6–37.5	90.4–91.9
CCoV-I-Elmo/02	AY307020	37.6–40.4	61.8–62.8
CCoV-I-23/03	AY307021	31.8–34.8	60.3–61.2
FCoV-I-KU-2	D32044	37.4–40.8	59.1–60.3
FCoV-I-Black	EU186072	36.2–39.3	58.9–61.0
FCoV-I-UCD1	AB088222	38.3–42.7	57.9–58.9

*TGEV, porcine transmissible gastroenteritis virus; CCoV, canine coronavirus; NA, sequence not available. †Nucleotides 1–305 of the spike gene of reference strain CCoV-IIb-341/05. ‡Nucleotides 3574–4085 of the spike gene of reference strain CCoV-IIb-341/05.

At the phylogenetic level, the sequenced strains were grouped in the same cluster with TGEV and prototype CCoV-IIb strains at the 5′ end of the spike protein gene, displaying an obvious distance to both CCoV-IIa/FCoV-II and CCoV-I/FCoV-I (Figure, panel A). The TGEV-like CCoVs from the United Kingdom formed a unique clade, whereas the strains detected in eastern Europe were mixed with CCoV-IIb viruses from Italy. The old TGEV-like strain, UCD1, clustered with TGEV isolates. At the 3′ end of the same gene, subtype IIa and IIb strains segregated together and were separated from the FCoVs and the porcine CoVs TGEV and PRCoV (Figure, panel B). In addition, the strains from the United Kingdom were again grouped in a separate subcluster.

**Figure:**
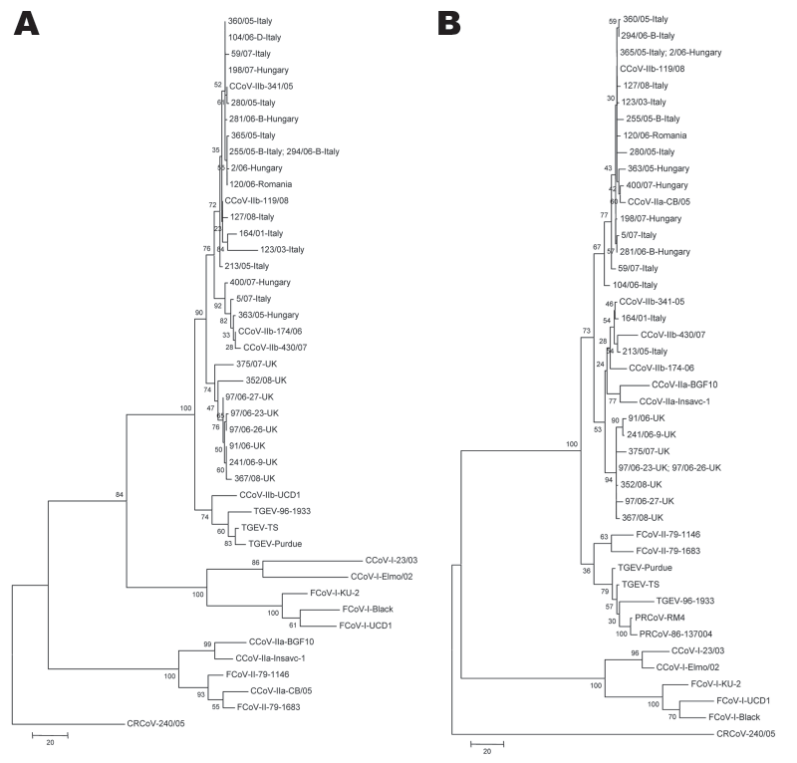
Phylogenetic analysis of canine coronavirus (CCoV) type IIb. Maximum parsimony trees based on partial 5´ (A) and 3´ (B) ends of the spike protein gene of group-1a coronaviruses (CoVs). For phylogenetic tree construction, the following reference strains were used (GenBank accession numbers are in parentheses): porcine transmissible gastroenteritis virus (TGEV) Purdue (NC_002306), TS (DQ201447), 96-1933 (AF104420), porcine respiratory coronavirus (PRCoV, only 3´ end) RM-4 (Z24675), 86-137004 (X60089), CCoV-IIa CB/05 (DQ112226), Insavc-1 (D13096), BGF10 (AY342160), CCoV-IIb 341/05 (EU856361), 174/06 (EU856362), 430/07 (EU924790), 118/08 (EU924791), UCD-1 (AF116248, only 5´ end), CCoV-I Elmo/02 (AY307020), 23/03 (AY307021), feline coronavirus (FCoV) type I Black (EU186072), KU-1 (D32044), UCD-1 (AB088222), FCoV-II 79-1146 (NC_007025), 79-1183 (X80799). The tree is rooted on the group-2 CoV canine respiratory coronavirus (CRCoV) 240/05 (EU999954). Statistical support was provided by bootstrapping >1,000 replicates. Scale bars indicate estimated numbers of nucleotide substitutions per site.

## Discussion

CoVs are exceptionally prone to variability through accumulation of point mutations and recombination events. A CCoV strain displaying close relatedness to porcine CoVs in the N-terminus of the spike protein, which is related to porcine CoVs, was isolated ≈20 years ago, but the subsequent molecular characterization was restricted to the 5′ end of the spike gene (*19*). Additional TGEV-like CCoVs were reported more recently in Italy (*14*) and the United Kingdom (*15*). However, full biological and molecular characterization was carried out only for the isolates from Italy, showing that the TGEV-like strains are likely recombinant with TGEV at the level of the 5′ end of the spike gene (*14*). Experimental infection of CCoV-seronegative beagle pups showed that TGEV-like (i.e., subtype IIb) CCoV induces clinical signs resembling those of classical (i.e., subtype IIa) CCoVs, that is, mild diarrhea for a few days. (*14*) Unlike pantropic CCoV (*11*–*13*), TGEV-like CCoV was not able to spread systemically. Of the 4 recombinant strains detected in Italy, 2 had originated from eastern Europe, but at the phylogenetic level they were mixed with strains from Italy. The prevalence of this CCoV subtype in the canine population has not been determined in previous studies.

Our epidemiologic investigation assessed the distribution of CCoV-IIb in the dog population of Europe. Approximately 50% of the analyzed samples were positive for CCoV, showing the presence of CCoV-I or CCoV-II. Mixed infections caused by both genotypes were detected in <40% of the CCoV-positive samples, considerably lower than previously reported percentages (*7*,*17*). Approximately 20% of the CCoV–II-positive samples contained TGEV-like strains (Table 1). However, the prevalence of this CCoV subtype differed by geographic origin of the samples; the highest detection rates (96.87%) were observed in Hungary. The recombinant origin of all strains characterized by RT-PCR was confirmed by sequence analysis of 5′ and 3′ ends of the spike gene and by RT-PCR of the ORF7a/7b region. The selected 26 TGEV-like strains were related to prototype strains from Italy (*14*) in both the 5′ and the 3′ ends of the spike gene. A comparison with the prototype UK strains reported by Erles and Brownlie (*15*) was not possible, however, because the unique spike sequence deposited in the GenBank is located more downstream of the gene with respect to the sequences that we obtained.

On the basis of the spike gene sequences, the strains from Italy and eastern Europe were closely related, whereas the strains from the United Kingdom were more genetically distant (Table 3). This pattern of segregation was confirmed by phylogenic analysis, which showed that viruses detected in the United Kingdom formed a separate cluster with respect to the samples from Italy, Hungary, and Romania (Figure). The genetic relatedness between the TGEV-like strains from Italy and those from eastern Europe may be accounted for by extensive dog importation to Italy. In addition, dog exchange between eastern Europe and Italy has been associated with the reemergence of canine infectious hepatitis (*27*) and the spread of the arctic lineage of canine distemper virus (*28*).

In the 5′ end of the spike gene, the old TGEV-like strain, UCD1, was found to be genetically more related to true TGEV isolates than to recent CCoV-IIb strains, thereby accounting for recombination events occurring at different times. With the exception of strain UCD1, analysis of archival samples found the oldest TGEV-like strain in 2001, about 4 years before this CCoV subtype was reported in Italy (*14*).

Our study confirms that recombinant CCoVs are effectively circulating in dogs in different European countries. Considering the genetic distance in the spike protein, this circulation questions the efficacy of vaccines, which are based on classical (CCoV-IIa) strains, against the emerging TGEV-like (CCoV-IIb) viruses. Only vaccination trials and subsequent challenges by TGEV-like strains might assess whether the poor cross-reactivity between CCoV-IIa and CCoV-IIb observed in a previous study (*14*) might affect the immune response of dogs against the recombinant viruses.
